# Magnitude and determinants of animal source food consumption among children aged 6–23 months in Ethiopia: secondary analysis of the 2016 Ethiopian demographic and health survey

**DOI:** 10.1186/s12889-022-12807-8

**Published:** 2022-03-07

**Authors:** Gebretsadkan Gebremedhin Gebretsadik, Amaha Kahsay Adhanu, Afework Mulugeta

**Affiliations:** Department of Nutrition and Dietetics, School of Public Health, College of Health Sciences, Mekelle University, Mekelle, Ethiopia

**Keywords:** ASF, Complementary food, Young children, DHS, Ethiopia

## Abstract

**Background:**

Undernutrition puts children in a physical and cognitive disadvantage. Animal source foods (ASFs) are important components of nutritious diets and play a significant role in increasing dietary diversity and minimizing the risk of undernutrition among children. Ethiopia still suffers from child undernutrition and there’s no adequate information regarding consumption of ASFs. The objective of this study was to determine the magnitude and determinanats of ASF consumption among children 6–23 months of age.

**Methodology:**

A total weighted sample of 2861 children drawn from the 2016 Ethiopian demographic and health survey was analyzed using “SVY” command of STATA 14.0. Multivariable logistic regression was used to determine the independent determinants of ASF consumption. The strength of the association was measured by odds ratio and 95% confidence interval and *p*-value < 0.05 was considered statistically significant.

**Results:**

Nearly half (46.5%) of the children reported consuming any type of ASF. Religion, child age, number of household assets, number of livestock owned by a household, and ownership of land usable for agriculture were significant determinants of the outcome variable. The odds of ASF consumption were six times, twice, and 70% lower in orthodox children compared to other (catholic, traditional, or others), muslim, and protestant children, respectively. Household ownership of assets and livestock led to an increase in consumption of ASF by 19 and 2%, respectively. Children aged 18–23 months were more likely to consume ASF as compared to the younger age group (6–8 months old children). In the contrary, children from households that own land usable for agriculture were 33% less likely to consume ASFs as compared to those from households that do not own.

**Conclusions:**

In Ethiopia, only nearly half of children aged 6–23 months consume any type of ASF. The findings of this study imply that ASF consumption can be increased through integrated actions that involve community and religious leaders and programs focused on empowering households’ capability of owning other socioeconomic entities including assets and livestock. This study also may contribute to the growing body of research works on the importance of ASF provision in preventing child undernutrition.

## Introduction

Child undernutrition, the damaging result of poor nutrition during the first 1000 days of life, puts children in a physical and cognitive disadvantage [1]. Globally, 149 million under 5 children were estimated to be stunted in 2018 [2]. Undernutrition has been known as an underlying cause for reduced cognitive skills, lower school performance, morbidities, poverty, and nearly half of under-five deaths [3]. Standard infant and young child feeding recommendations have been established as ways to improve nutrition outcomes [4]. At 6 months of age, infants should start complementary feeding without stopping to breastfeed. A highly diversified complementary diet increases energy and nutrients intakes, and hence improves the nutritional status of children [5].

Animal-source foods (ASFs) are important components of nutritious diets and play a significant role in minimizing the risk of undernutrition among vulnerable groups [6]. Consumption of such foods by older infants and young children has been found to increase dietary diversity. High Dietary diversity has been shown to strongly contribute to improved nutritional results in children [7]. According to a systematic review of studies done in food-insecure areas, consumption or provision of additional complementary foods has been effective at reducing undernutrition. However, to get better results, this should be combined with education [8, 9]. Dairy products (milk, yogurt, cheese), flesh foods (meat, fish, poultry and organ meats), and eggs are the three ASFs that make-up the eight food groups illustrated by the World health organization (WHO) to determine optimal dietary diversity [10].

Animal source foods (ASFs) are energy-dense foods that contain the highest quality proteins yielding all the essential amino acids in amounts and forms human beings require. They are also efficient sources of bioavailable micronutrients including iron, zinc, vitamin A, vitamin B12, riboflavin, and calcium, which are of utmost concern globally when addressing food insecurity [11, 12]. Besides, they contain lower levels of anti-nutritional factors compared to plant source foods [13].

Although the stunting reduction rate Ethiopia has achieved is outstanding, the problem remains a major challenge, with approximately two out of five under 5 children are stunted (short for their age). Moreover, micronutrient deficiencies, especially of iron, vitamin A, iodine and zinc, are common among children. Annually, such problems of undernutrition cost the country 4.7 billion USD or 16% of its Gross domestic product (GDP). To tackle such problems of undernutrition, Ethiopia developed and applied different approaches like the National Nutrition Program [14] and a recent Food and Nutrition Policy [15].

In low and middle-income countries like Ethiopia, access to and availability of ASFs is usually limited for many children. According to the Ethiopian demographic and health survey (EDHS), only 14% of children aged 6–23 months meet the minimum dietary diversity requirements. This report noted that only 8% of children consumed meat, fish, or poultry. It also showed that about 17 and 25% of children aged 6–23 months reported consumption of eggs and dairy products, respectively [16]. A study conducted in Tigray region also found that only 13% of the children 6–23 months old meet the WHO recommended dietary diversity [17]. One reason for the diets being extremely monotonous could be poor consumption of ASFs.

Various factors may influence the availability and consumption of ASFs by children. Factors that were associated with increased ASF consumption include increased child age, pastoral livelihood, Muslim religion, and participation in a Productive Safety Net Program [18]. Besides, villages in forested areas with wild animal populations, observing a large number of ceremonies of long duration, households with a greater number of small livestock, and women’s autonomy on decisions about livestock assets also were related to increased ASF intake [19].

Information regarding the magnitude and determinants of ASF consumption among children is inadequate at national level. Ethiopia is a country with diverse livelihoods, religions, and cultures that can affect consumption of ASFs among children. In an area with such socioeconomic and cultural diversity, much research on this topic is necessary to provide more conclusive information to establish effective interventions to improve dietary diversity and reduce stunting.

Therefore, with this study, we aimed to fill the gap of information regarding the consumption of ASF and the factors associated with it at the national level. The objective of this study was to determine the magnitude and factors associated with ASF consumption among children aged 6 to 23 months in Ethiopia.

## Methodology

### Data source and sample design

The analysis for this study was based on data extracted from the 2016 Ethiopia Demographic and Health Survey (2016 EDHS), which is the fourth in a series of Demographic and Health Surveys conducted in Ethiopia. This survey used a sampling frame of a complete list of 84,915 enumeration areas (EAs) created for the 2007 Ethiopia Population and Housing Census. An EA covers on average 181 households. The survey involved a two-stage stratified sampling. Each of the 11 administrative regions in the country was stratified into urban and rural areas, which resulted in 21 sampling strata. The first stage involved selection of 645 EAs with probability proportional to EA size and with independent selection in each sampling stratum. In the second stage, from the new hosehold list, a fixed number of 28 households per cluster were selected with an equal probability systematic selection.

Among the total 18,008 households selected for the sample, 17,067 were available during data collection, and 16,650 were successfully interviewed, giving a response rate of 98%. Among the 16,583 eligible women for individual interviews in the interviewed households, only 15, 683 were successful yielding a response rate of 95%. Women who had no less than one child living with them who was born in 2014 or later were asked questions about the types of liquids and foods the child had consumed in the 24 h before the survey. If mothers had more than one child aged 6–23 months, they were interviewed only about the youngest one [20]. Our analysis was done among last born living children aged 6–23 months that live with the respondent.

### Study variables

#### Outcome variable

The outcome variable for this study was consumption of ASF by children aged 6–23 months. It was categorized into “0”(No ASF consumption) and “1”(ASF consumption). The questionnaire asked mothers/caretakers the types of foods the child had eaten in the 24 h prior to the survey. Eggs, fish, yogurt, cheese, milk, meat (including beef, poultry, pork, lamb, and any other meat not mentioned), and organ meats (e.g., liver) were the ASFs included in the questionnaire. Consumption of any amount and/or type of the ASFs listed above was considered as ASF consumption.

#### Explanatory variables

Control variables were selected based on their hypothesized effect on the outcome variable and their availability in the data. Variables included in this analysis were child sex and age, respondent age, respondent educational status, respondent’s occupation, household total number of children, household assets, household wealth index, access to media at least once a week, religion, ownership of land usable for agriculture, and total number of livestock owned.

Child age was categorized into four groups (6–8 months, 9–11 months, 12–17 months, and 18–23 months). Respondent’s age was also a factor variable with three levels (15–24 years, 25–34 years, and 35–49 years). Educational status of respondents was dichotomized into no and any education. Ownership of household assets was determined from a summed score of a set of twelve assets including electricity, a watch or clock, a radio, a television, a mobile telephone, a non-mobile telephone, a refrigerator, a table, a chair, a bed with a mattress (cotton/sponge/spring), an electric mitad (a grill or cooktop used for preparing injera or bread), and a kerosene lamp/pressure lamp. Access to media was also dichotomized into exposure to media (reading newspaper, listening to radio, or watching television) at least once a week or not. Livestock ownership was taken into account if a household owns any livestock (cattle, cows/bulls, goat, sheep, chicken, or camel). Moreover, occupational status of respondents was categorized into three categories (Not working, agricultural works, and non-agricultural works).

### Statistical analysis

Data analysis was done using Stata version 14.0 after the complex sampling design was adjusted by applying sampling weights using the survey “SVY” command. Sampling weights were used to remove the probability of including an observation that would happen due to the complex sampling design and avoid potential bias. Before starting data analysis, data cleaning and selection of appropriate control variables were performed. Descriptive characteristics are presented as proportions (%) and means for categorical and continuous variables, respectively.

Multivariable logistic regression was then used to calculate adjusted odds ratios (OR) and 95% confidence intervals (CI). Variables that satisfied the cutoff point of *p-*value ≤0.25 in the bivariate model were candidates for the multiple logistic regression model. Finally, only those variables with statistical significance of *p* < 0.05 were retained in the final model. Hosmer and Lemeshow goodness-of-fit test was used to test model fitness. Multi-collinearity among independent variables was also assessed using variance inflation factor (VIF) and a value of 10 was used as cut off.

## Results

### Participant characteristics

Among the 2861 children in the sample, 50.4% were females and the mean age was 13.9 ± 5.1 months. Majority (87.9%) of the respondents lived in rural areas. The average age of parents/caregivers was 28.6 ± 6.6 years and about one-third reported having attained any formal education. About 40.2 and 34.4% of the surveyed households were Muslim and Orthodox Christians, respectively. Concerning educational status, 38.8% of the mothers/caregivers and 43.1% of the husbands received any form of education. Agricultural works represented 52 and 64% of the occupations of the mothers/caregivers and fathers of the children, respectively. The average number of assets a surveyed household-owned was 2.86 (out of potential 8). More than three quarters (77%) of the households owned land usable for agriculture. Similarly, about 73.4% of the surveyed households owned livestock. This study showed low exposure of mothers/caregivers to mass media. From the interviewed mothers/caregivers 80.7% have access to media less than once a week or do not have any kind of access to media at all. (Table 1).

**Table 1:** Household, caretaker, and child characteristics of children aged 6–23 months in Ethiopia, *n* = 2861

Variable (Missing)		Frequency (%)^a^
Place of residence (0)	Urban	601 (12.1)
	Rural	2260 (87.9)
Respondent’s age in years	Mean (SD)	28.6 (6.6)
	Median (Q1, Q2)	28 (24, 33)
Respondent age in years	15–24	856 (27.5)
	25–34	1448 (52.0)
	35–49	557 (20.5)
Religion (0)	Orthodox	864 (34.4)
	Muslim	1418 (40.2)
	Protestant	509 (22.0)
	Others	70 (3.4%)
Respondent education (0)	No education	1715 (61.2)
	Any education	1146 (38.8)
Husband education (146)	No education	1799 (58.7)
	Any education	916 (41.3)
Number of under 5 children (0)	Mean (SD)	1.8 (0.8)
	Median (Q1, Q3)	2 (1, 2)
Wealth index (0)	Poorest	963 (23.2)
	Poorer	479 (21.2)
	Middle	426 (22.0)
	Richer	352 (18.2)
	Richest	641 (15.4)
Caretaker occupation (0)	Not working	1044 (26.9)
	Agricultural	1231 (52.0)
	Non-agricultural	586 (21.1)
Husband occupation (146)	Not working	311 (8.3)
	Agricultural	1407 (64.0)
	Non-agricultural	997 (27.7)
Access to media at least once a week	No	2223 (80.7)
	Yes	638 (19.3)
Total number of livestock (0)	Mean (SD)	8.9 (11.8)
	Median (Q1, Q3)	6 (2, 12)
Livestock ownership (0)	Does not own	762 (17.5)
	Own	2099 (82.5)
Ownership of land usable for agriculture (0)	Does not own	1077 (23.0)
	Own	1784 (77.0)
Number of household assets (0)	Mean (SD)	2.7 (2.4)
	Median (Q1, Q3)	2 (1, 4)
Child sex	Male	1419 (47.1)
	Female	1442 (52.9)
Child age in months	Mean (SD)	13.9 (5.0)
	Median (Q1, Q3)	14 (10, 18)
Minimum dietary diversity	No (< 4 food groups	2491 (86.3)
	Yes (> = 4 food groups)	370 (13.7)

^a^For continuous variables, mean (SD) and median (Q1,Q3) are presented. *SD* Standard deviation, *Q1* first quartile, *Q3* Third quartile

Table 1: Household, caretaker, and child characteristics of children aged 6–23 months in Ethiopia, *n* = 2861.

### ASF consumption

**Fig. 1:**
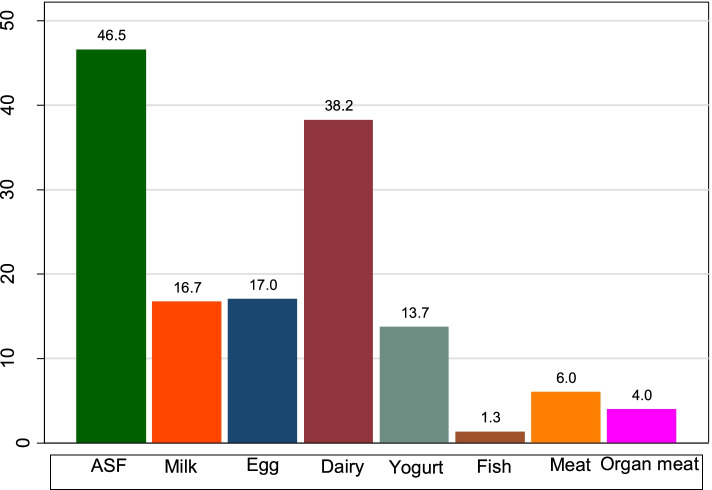
Consumption of specific ASFs among children aged 6–23 months in Ethiopia, n = 2861. ASFs, animal source foods

In the presented study, 46.5% (42.9, 50.2) children reported consuming any type of ASF. Besides, 38.2% (34.7, 41.9) reported consuming dairy (includes tinned, powdered or fresh milk, cheese, yogurt, or other milk products). Among the total number of children in the sample, 17% (95% CI 14.9, 19.4), 16.7% (95% CI 14.4, 19.3), and 13.7% (95% CI 11.6, 16.2) consumed egg, milk, and yogurt in the 24 h preceding the survey, respectively. Consumption of meat (6%) (95% CI 4.3, 8.4), organ meat (4%) (95% CI 3.0, 5.2, and fish (1.3%) (95% CI 0.6, 3.1) was very low (Fig. 1).

**Fig. 2:**
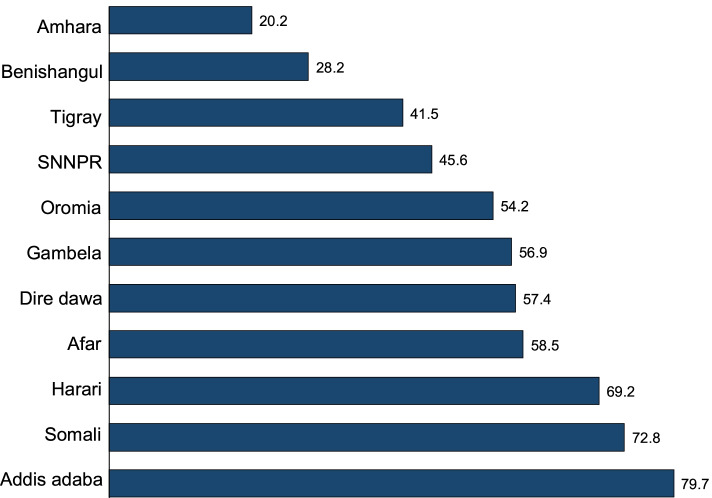
Consumption of ASFs among children aged 6–23 months in Ethiopia by region, *n* = 2861. ASFs, animal source foods

Consumption of ASF showed regional differences. Addis Ababa (79.7%), Somali (72.8%), and Harari (69.2%) regions had the highest proportions of children consuming ASFs. ASF consumption was low in Amhara (20.2%) and Benshangul-gumuz (28.2%) regions (Fig. 2).

About 50.9% of children from Muslim households consumed ASF. This is higher compared to that of children from orthodox (37.8%), and protestant (47.7%). However, among children who fall under the “Others” (*n* = 70) religion, 75% consumed ASF in the previous day of the survey. Children from mothers who obtained any education consumed ASF at higher rates (53.1%) than those from mothers with no education (42.4%). Besides, ASF consumption was higher in children from respondents that had access to media at least once a week (58.1%) in contrast to those from respondents that have access to media less than once a week or have not access at all (43.8%). Moreover, the mean number of assets and livestock owned by a household were both greater in the group consuming ASF.

### Determinants of ASF consumption

The variables that passed the screening (*p*-value< 0.25) in the bivariate analysis include place of residence, religion, total number of household assets, total number of livestock owned by a household, ownership of land usable for agriculture, child age, child sex, age of respondent, educational status of the respondent, and respondent’s access to media at least once a week. Finally, five variables made up the final adjusted logistic regression model as determinants of ASF consumption (*p*-value< 0.05). (Table 2).

**Table 2:** Bivariate and multivariate analysis of factors associated with ASF consumption among children aged 6–23 months in Ethiopia, *n* = 2861

Variables		ASF Consumption			COR (95%CI)	*P*-value	AOR(95%CI)	*P*-value
		No	Yes	Total				
Place of residence	Urban (Ref)	212	389	601	1			
	Rural	1168	1092	2260	0.47 (0.33,0.68)	< 0.001	0.88 (0.50,1.54)	0.658
Religion	Orthodox (Ref)	502	362	864	1			
	Muslim	603	815	1418	1.7 (1.24, 2.34)	0.001	2.26 (1.58,3.23)	< 0.001**
	Protestant	246	263	509	1.5 (1.07, 2.1)	0.019	1.70 (1.16,2.46)	0.005**
	Others	29	41	70	4.94 (1.45,16.82)	0.011	5.80 (1.64,20.17)	0.006**
Wealth index	Poorest (ref)	453	510	963	1			
	Poorer	285	194	479	0.79 (0.5, 1.21)	0.279	0.87 (0.61,1.25)	0.451
	Middle	225	201	426	1.01 (0.65, 1.59)	0.936	0.99 (0.69,1.42)	0.948
	Richer	183	169	352	1.07 (0.67, 1.67)	0.789	0.78 (0.51,1.20)	0.264
	Richest	234	407	641	2.03 (1.26, 3.30)	0.003	0.90 (0.46,1.79)	0.772
Respondent education	No education (Ref)	896	819	1715	1			
	Any education	484	662	1146	1.54 (1.19,1.98)	0.001	1.29 (0.97,1.71)	0.076
Respondent age	15–24 (Ref)	401	455	856	1			
	25–34	674	774	1448	0.97 (0.74,1.27)	0.823	0.97 (0.73,1.27)	0.803
	35–49	305	252	557	0.71 (0.52,0.97)	0.030	0.79 (0.56,1.11)	0.174
Number of household assets	Mean (SD)	2.2 (2.0)	3.2 (2.6)	2.7 (2.4)	1.19 (1.13,1.26)	< 0.001	1.19 (1.09,1.30)	< 0.001**
Number of livestock	Mean (SD)	8.1 (10.2)	9.8 (13.3)	8.9 (11.8)	1.01 (1.01, 1.02)	0.022	1.02 (1.01,1.03)	< 0.001**
Ownership of land usable for agriculture	No (Ref)	416	661	1077	1			
	Yes	964	820	1784	0.57 (0.44,0.72)	< 0.001	0.67 (0.51,0.89)	0.006**
Child age in months	6–8 (ref)	308	231	539	1			
	9–11	198	258	456	1.54 (1.12,2.10)	0.008	1.61 (1.13,2.28)	0.008**
	12–17	505	581	1086	1.33 (1.01,1.78)	0.049	1.44 (1.06,1.95)	0.019*
	18–23	369	411	780	1.36 (1.01,1.84)	0.047	1.53 (1.11,2.12)	0.009**
Child sex	Male (ref)	647	772	647	1			
	Female	733	709	733	0.79 (0.64, 0.98)	0.029	0.78 (0.62,0.98)	0.060
Access to media at least once a week	No (ref)	1143	1080	2223	1			
	Yes	237	401	638	1.78 (1.29,2.46)	< 0.001	0.95 (0.64,1.39)	0.780

*p < 0.05, **p < 0.01, *ASF* Animal source foods, *SD* Standard deviation

Compared to children from an orthodox family, the odds of ASF consumption were about six times, twice, and 70% higher in children from households with other (catholic, traditional, or others) (AOR = 5.80; 95% CI 1.64, 20.17), Muslim (AOR = 2.26; 95% CI 1.58, 3.23), and protestant (AOR = 1.70; 95% CI 1.16, 2.46) religions, respectively. Besides, the average number of household assets (AOR = 1.19; 95% CI 1.09, 1.30) and the average number of livestock owned by a household (AOR = 1.02; 95% CI 1.01, 1.03) were significant determinants of ASF consumption in the analyzed sample. Moreover, higher odds of ASF consumption were seen in children aged 9–11 months (AOR = 1.61; 95% CI 1.13, 2.28), 12–17 months (AOR = 1.44; 95% CI 1.06, 1.95), and 18–23 months (AOR = 1.53; 95% CI 1.11, 2.12) in contrast to the younger age group (6–8 months old children). Opposed to the positive associations described above, one variable was found to be negatively associated with the outcome variable. Children from households that own land usable for agriculture were 33% (AOR 0.67; 95% CI 0.51, 0.89) less likely to consume ASFs as compared to those from households that do not own land usable for agriculture. (Table 2).

**Fig. 3:**
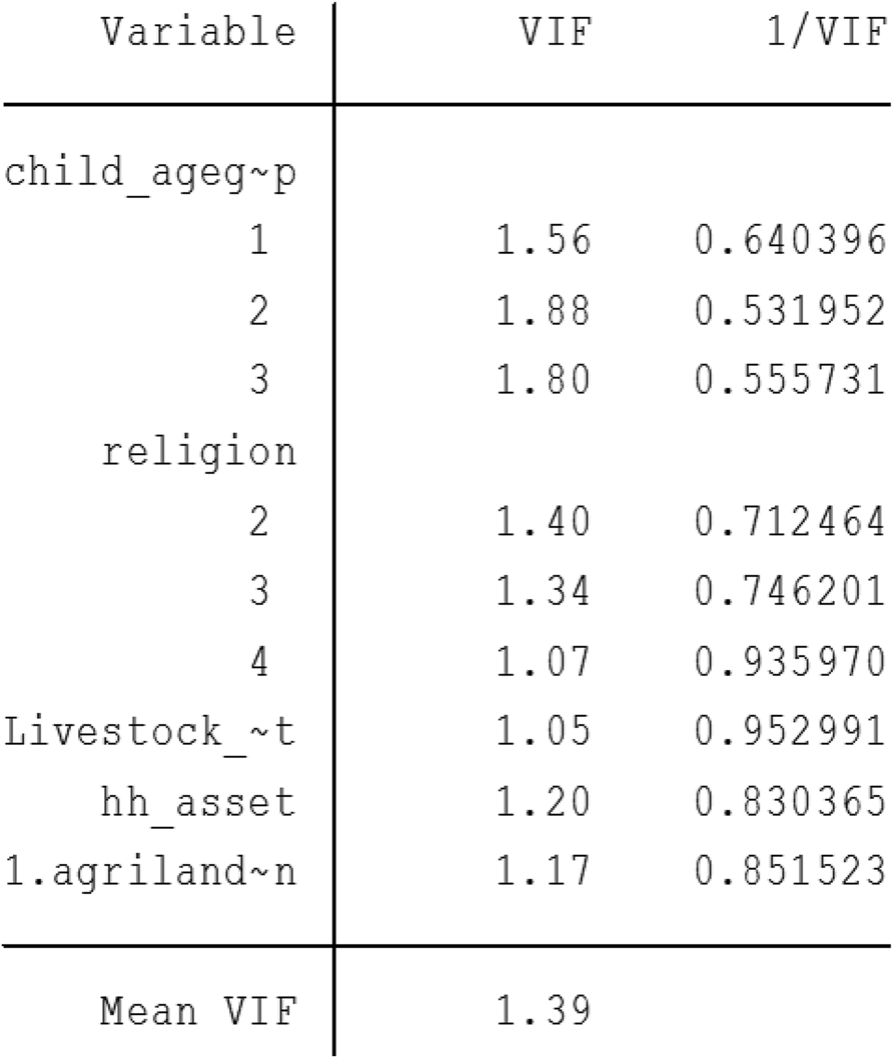
Multicollinearity test for predictors in the final model of ASF consumption among children aged 6–23 months in Ethiopia, *n* = 2861. VIF, Variance inflation factor

The final model of this study indicated that there was no multicollinearity shown by a mean VIF value of 1.39. (Fig. 3). The significance level of Hosmer–Lemeshow test for goodness of fit was 0.34. Since this value is greater than 0.05, we fail to reject the null hypothesis and this shows that there is no significant difference between the observed and model predicted values. Therefore, our final model fits the data well.

## Discussion

In this secondary analysis of the 2016 EDHS, which included a total of 2861 children aged 6–23 months, 46.5% reported consuming any type of ASF in the day prior to the survey. This is similar to a recent survey in Ethiopia that found a slightly higher magnitude (51%) of ASF consumption [18]. A survey in Kenya reported that less than 30 and 10% of children consumed flesh foods and eggs, respectively [21]. The lower magnitude in the latter study could be due to the absence of information on dairy consumption.

Religion, number of household assets, number of livestock owned by a household, ownership of land usable for agriculture, and child age are the five variables that were significantly associated with the outcome variable.

Children from orthodox family had lower odds of ASF consumption compared to children from other religions. Not differently, a study that assessed ASF consumption by children from four regions of Ethiopia found a 30% lower consumption in children from Orthodox households than children from Muslim households [18]. Throughout the year, Ethiopian Orthodox Christians have seven official fasting periods; All Wednesday and Fridays, except for the 50 days after Easter, the Lenten fast of 55 days, the Nineveh fast of 3 days, the vigils, or gahad of Christmas and epiphany, the 14–44 days fast of the apostles, the 43 days of the fast of the prophets, and the 15 days fast of the assumption in august. The average person may fast about 180 days, refraining from consuming ASF including meat, eggs, and milk [22]. During these extended time periods, a person is expected not to consume any food of animal origin. Although children are not supposed to fast, the limited availability of ASFs in the households they live during such periods may affect their consumption of those foods. The other reason that could affect ASF consumption by children is fear of contamination of cooking utensils and violation of religious rules and procedures by caregivers [23]. Notwithstanding the above explanations, a recent report by Alive and Thrive on IYCF practices, beliefs, and influences in Tigray region discussed that some mothers reported unaffordability and unavailability of meat and chicken in the market in fasting periods [24]. This could be due to the fact that ASFs especially meat are scarce and more expensive particularly during long periods of fasting like Lent in Orthodox communities because fasting adults are unwilling to slaughter animals [25].

Besides, children from households with higher numbers of livestock showed higher ASF consumption. Other studies found similar findings. Compared to households that did not own a particular livestock group, consumption of ASF during the 30 days before the survey was found to be significantly higher in households that owned the livestock group from which the food was derived [26]. Besides, according to a study in rural Uganda, promoting (small) livestock ownership significantly increased dairy consumption [27]. Moreover, in a study in rural Nepal, even low levels of poultry and cattle ownership were respectively associated with higher consumption of eggs and dairy among young children [28]. Ownership of livestock is also a positive facilitator of child dietary diversity [29]. These findings support the argument that households are utilizing livestock for own consumption. Therefore, such a positive association between livestock ownership and ASF consumption should be maintained by addressing socio-cultural norms and practices towards motives of livestock keeping, promoting good livestock rearing practices, improving market access, and filling gaps on nutrition importance of ASFs [30]. This is especially important in Ethiopia, a country known as one of the owners of huge number of livestock in the world [31].

In contrast to the findings in our study, a study by Kobina et al. noted that household ownership of any livestock, but not poultry, was associated with decreased consumption of ASFs [32]. The reason for this difference could be the higher ownership of free-range poultry compared to other livestock and the relatively very low sample size in the Kobina et al. study. Similarly, a study in Luangwa Valley, Zambia, found no significant association between livestock ownership and ASF consumption [33].

In this presented study, ASF consumption increased by nearly 20% with a unit increase in the number of household assets. This is similar to a study done in Indonesia in which increased value of household assets was significantly associated with ASF consumption [34]. Economic capability of households possesses a great role in determining the likelihood of ASF consumption [35]. Households with a higher number of assets (electricity, a television, a radio, mobile/non-mobile telephone, a refrigerator, a table, a chair, a bed with a mattress (cotton/sponge/spring), an electric mitad (a grill or cooktop used for preparing injera or bread), and a kerosene lamp/pressure lamp) may have a better opportunity to prepare and present ASFs on their table for consumption.

This study showed higher odds of ASF consumption among children in higher age categories. Similarly, in their study about ASF and child stunting, Headey et al. witnessed that consumption of any ASF and only dairy (consumption of meat, eggs, and fish were similar across age groups) increased with age [36]. The reason could be that as age is increasing, it is more likely feeding skills are improved and caloric needs are increased.

Nevertheless, children from households that own land usable for agriculture showed lower ASF consumption. This is challenged by a recent report by food and agriculture organization which states that the ownership, including its quality, of agricultural land is a major positive determinant of the relationship between crop and livestock production [37]. The reason for this negative association could be the fact that more than 72% of households that own land usable for agriculture in this study lie within the three lowest wealth quintles (middle, poorer, and poorest). These households are more likely to sell and/or exchange any of their livestock for other necessary goods than using for own consumption. In such households, ASFs are considered as luxury foods and usually are only consumed during holidays and special events.

### Strengths and limitations of the study

The main strength of this study is that it utilized a large sample size and that the sampling technique (especially the use of sampling weights) allowed every household to have equal probability of inclusion. The larger sample size proved important in maintaining the internal validity of the study by helping provide precise descriptive and analytic findings. The other strength is that the data in this study were collected from all the regions in Ethiopia, which makes generalizability of the findings forward.

The crosssectional design of this study means it doesn’t show any cause and effect relationship between the outcome variable and the explanatory variables. Additionally, lack of information on possible explanatory variables like food insecurity status of the households may have changed the picture of the current associations between the variables. Therefore, incorporating a household food insecurity access score in future studies can be crucial in identifying other unseen associations. Another possible limitation is that this study did not provide information on the amount and frequency of ASF consumption. The use of 24-h dietary recalls may not perefectly tell about the usual diets (i.e., by creating within-person error) leading to an attenuation bias [38]. Therefore, future similar studies should consider and try to address the points raised.

## Conclusions

In Ethiopia, consumption of ASFs among children aged 6–23 months is not adequate. Besides, child age, religion, household livestock ownership, household ownership of assets, and household ownership of agricultural land determine ASF consumption among children. The findings of this study imply that ASF consumption can be increased through integrated work on community factors like religion and programs focused on empowering households’ capability of owning livestock and other socioeconomic assets. This study may contribute to the growing body of research works about ASF provision to older infants and young children.

## Data Availability

Supporting data for the current study are available from the corresponding author upon a reasonable request. The EDHS data was retrived from https://dhsprogram.com/data/available-datasets.cfm

## Abbreviations

AOR: Adjusted odds ratio
ASF: Animal source food
CI: Confidence interval
COR: Crude odds ratio
EA: Enumeration areas
EDHS: Ethiopian demographic and health surveys
DHS: Demographic and health surveys
GDP: Gross domestic product
OR: Odds ratio
SD: Standard deviation
SVY: Survey
VIF: Variance inflation factor
WHO: World health organization
